# Association of Angiopoietin-2 with Renal Outcome in Chronic Kidney Disease

**DOI:** 10.1371/journal.pone.0108862

**Published:** 2014-10-03

**Authors:** Yi-Chun Tsai, Yi-Wen Chiu, Jer-Chia Tsai, Hung-Tien Kuo, Su-Chu Lee, Chi-Chih Hung, Ming-Yen Lin, Shang-Jyh Hwang, Mei-Chuan Kuo, Hung-Chun Chen

**Affiliations:** 1 Graduate Institute of Clinical Medicine, Kaohsiung Medical University, Kaohsiung, Taiwan; 2 Division of Nephrology, Department of Internal Medicine, Kaohsiung Medical University Hospital, Kaohsiung, Taiwan; 3 Faculty of Renal Care, College of Medicine, Kaohsiung Medical University, Kaohsiung, Taiwan; 4 Institute of Population Sciences, National Health Research Institutes, Miaoli, Taiwan; National Center for Scientific Research Demokritos, Greece

## Abstract

**Background:**

The pathophysiological mechanisms of renal function progression in chronic kidney disease (CKD) have still not been completely explored. In addition to well-known traditional risk factors, non-traditional risk factors, such as endothelial dysfunction, have gradually attracted physicians' attention. Angiopoietin-2 (Ang-2) impairs endothelial function through preventing angiopoietin-1 from binding to Tie2 receptor. Whether Ang-2 is associated with renal function progression in CKD is unknown.

**Methods:**

This study enrolled 621 patients with stages 3–5 CKD to assess the association of circulating Ang-2 with commencing dialysis, doubling creatinine and rapid decline in renal function (the slope of estimated glomerular filtration rate (eGFR) greater than 5 ml/min per 1.73 m^2^/y) over follow-up of more than 3 years.

**Results:**

Of all patients, 224 patients (36.1%) progressed to commencing dialysis and 165 (26.6%) reached doubling creatinine. 85 subjects (13.9%) had rapid decline in renal function. Ang-2 quartile was divided at 1494.1, 1948.8, and 2593.1 pg/ml. The adjusted HR of composite outcomes, either commencing dialysis or doubling creatinine was 1.53 (95% CI: 1.06–2.23) for subjects of quartile 4 compared with those of quartile 1. The adjusted OR for rapid decline in renal function was 2.96 (95% CI: 1.13–7.76) for subjects of quartile 4 compared with those of quartile 1. The linear mixed-effects model shows a more rapid decrease in eGFR over time in patients with quartile 3 or more of Ang-2 than those with the lowest quartile of Ang-2.

**Conclusions:**

Ang-2 is an independent predictor of adverse renal outcome in CKD. Further study is needed to identify the pathogenic role of Ang-2 in CKD progression.

## Introduction

Chronic kidney disease (CKD) has been recognized as a worldwide health issue [1]. The pathophysiological mechanisms of renal function progression in CKD have still not been completely explored. In addition to well-known traditional risk factors, non-traditional risk factors, such as endothelial dysfunction, which might lead to cell apoptosis, vascular regression and renal fibrosis, have gradually attracted physicians' attention [2].

The angiopoietin (Ang)/Tie ligand-receptor system tightly controls the endothelial phenotype during angiogenesis and vascular inflammation [3]. Among the members of Ang family, Ang-1 and Ang-2 have attracted much attention [4]. Ang-1-driven Tie2 phosphorylation maintains structure integrity of vasculature, and protects the endothelium from excessive activation by cytokines and growth factors [5]. On the other hand, Ang-2 is expressed in endothelial cells, and stored in Weibel-Palade bodies (WPB) [6]. The rapid release of Ang-2 from endothelial cells upon activation of the endothelium by hypoxia, histamine, and thrombin would disrupt the protective, constitutive Ang-1/Tie2 signaling by preventing Ang-1 from binding to the receptor [5], [7]. Consequently, the loss of Tie2 signaling destabilizes the endothelium and contributes to angiogenic or inflammatory response to cytokines and growth factors [8].

Increased circulating Ang-2 has been found in diabetes mellitus [9], arterial hypertension [10], congestive heart failure [11], peripheral artery disease [12], coronary artery disease [13], sepsis [14], critical illness [15], and acute kidney injury [16]. Additionally, accumulating evidence shows that circulating Ang-2 is also markedly elevated in CKD and dialysis patients [17]. Elevated Ang-2 levels are also correlated with long-term mortality in patients with CKD stage 4 and on dialysis [18]. Although Ang-2 is associated with microalbuminuria [19], a clinical marker of renal injury, the relationship between Ang-2 and renal progression has not been well-explored in CKD patients not on dialysis. This study tries to analyze whether Ang-2 is associated with renal outcome, including reaching commencing dialysis and rapid decline in renal function (estimated glomerular filtration rate (eGFR) decline per year), in patients with CKD stages 3–5.

## Materials and Methods

### Study Participants

This observational study was conducted at a tertiary hospital in Southern Taiwan. Six hundred and twenty-one patients with CKD stages 3–5, who had follow-up for one year at least in our integrated CKD program, were enrolled in the study from January 2006 to December 2011. CKD was staged according to K/DOQI definitions and the eGFR was calculated using the equation of the 4-variable Modification of Diet in Renal Disease (MDRD) Study (CKD stage 3, eGFR: 30–59 ml/min/1.73 m^2^; CKD stage 4, eGFR: 15–29 ml/min/1.73 m^2^; CKD stage 5, eGFR <15 ml/min/1.73 m^2^) [20].

### Ethics Statement

The study protocol was approved by the Institutional Review Board of the Kaohsiung Medical University Hospital (KMUH-IRB-990198). Informed consents were obtained in written form from patients and all clinical investigations were conducted according to the principles expressed in the Declaration of Helsinki.

### Data Collection

Demographic and clinical data were obtained from medical records and interviews with patients at enrollment. The participant was asked to fast for at least 12 hours before blood sample collection for the biochemistry study and protein in urine was measured using urine protein-creatinine ratio. Patients were classified as diabetic by history and blood glucose values using the American Diabetes Association criteria, oral hypoglycemia agent use, or insulin use. Hypertension was defined as those with a history, or antihypertensive drugs use. Heart disease was defined as a history of heart failure, acute or chronic ischemic heart disease, or myocardial infarction. Cerebrovascular disease was defined as a history of cerebral infarction or hemorrhage. Information regarding patient medications including β-blocker, calcium channel blockers, angiotensin converting enzyme inhibitors (ACEI), and angiotensin II receptor blockers (ARB) before and after enrollment was obtained from medical records.

### Quantification of circulating Angiopoietin-2

Plasma Angiopoietin-2 was measured in duplicate using commercial enzyme-linked immunosorbent assays (R&D Systems Inc, Minneapolis, MN) according to the instructions of the manufacturer. The sensitivity of Ang-2 assay was 1.20 pg/ml. Intraassay and interassay coefficients of variation of Ang-2 were 1.8% and 1.2%, respectively.

### Renal Outcomes

Patients were contacted at outpatient clinics at 3-month intervals to ascertain the clinical status. Renal outcomes included commencing dialysis, doubling creatinine and rapid decline in renal function. Commencing dialysis was defined as requiring maintenance hemodialysis and peritoneal dialysis and confirmed by reviewing medical charts or catastrophic illness certificate (issued by the Bureau of National Health Insurance in Taiwan). The timing for commencing dialysis was considered according to the regulations of the Bureau of the National Health Insurance of Taiwan regarding eGFR, uremic status, nutritional status, and the laboratory data. The timing for doubling creatinine was considered based on all creatinine values from enrollment to the end of the observation period. The decline in renal function was assessed by the eGFR slope, defined as the regression coefficient between eGFR and time in units of ml/min per 1.73 m^2^ per year. All eGFR values available from enrollment to the end of the observation period were included for calculation. At least three eGFR values were required to estimate the eGFR slope. Rapid decline in renal function was defined as the eGFR slope greater than 5 ml/min/1.73 m^2^ per year based on Kidney Disease: Improving Global Outcomes (KDIGO) suggestion [21]. Patients were censored at death, last contact, or the end of observation in October 2013.

### Statistical Analysis

Baseline characteristics of all subjects were stratified by quartiles of Ang-2, cut at 1494.1, 1948.8, and 2593.1 pg/ml. Continuous variables were expressed as mean ±SD or median (25^th^, 75^th^ percentile), as appropriate, and categorical variables were expressed as percentages. Skewed distribution continuous variables were log-transformed to approximate normal distribution. The significance of differences in continuous variables between groups was tested using one-way analysis of variance (ANOVA) or the Kruskal-Wallis H test, as appropriate. The difference in the distribution of categorical variables was tested using the Chi-square test. Kaplan-Meier survival analysis was used to test Ang-2 as a predictor of the risk of composite outcomes either commencing dialysis or doubling creatinine. Cox regression models were applied to examine the relationship between Ang-2 and composite outcomes either commencing dialysis or doubling creatinine. Multivariable logistic regression models were also used to evaluate the association of Ang-2 with rapid decline in renal function. A linear mixed-effects model analysis was used to identify the factors associated with a change of eGFR, with control for internal correlations and other covariates. All the variables in Table 1 were tested by univariate analysis and those variables with P-value less than 0.05, including diabetes, heart disease, eGFR, urine protein-creatinine ratio cut at 1 g/g, serum albumin, phosphate, calcium, hemoglobin and cholesterol levels, and age, gender, and ACEI/ARB use were selected for multivariate cox and logistic analyses and linear mixed-effects model analysis. Statistical analyses were conducted using SPSS 18.0 for Windows (SPSS Inc., Chicago, Illinois). Statistical significance was set at a two-sided p-value of less than 0.05.

**Table 1:** The clinical characteristics of study subjects stratified by angiopoietin-2 quartile.

		Angiopoietin-2^a^				
	Entire Cohort N = 621	Quartile 1 N = 151	Quartile 2 N = 157	Quartile 3 N = 157	Quartile 4 N = 156	P-trend
Demographics						
Age (year)	65.3±12.7	62.2±13.6^#^	67.1±11.0*	65.9±12.2	66.0±13.2	0.005
Sex (male), n(%)	344(55.4)	98(64.9)	89(56.7)	83(52.9)	74(47.4)*	0.01
Smoke, n(%)	120(19.4)	36(23.8)	22(14.0)	26(17.0)	36(23.1)	0.1
Alcohol,n(%)	49(7.9)	20(13.2)^†^	13(8.3)	7(4.6)*	9(5.8)	0.03
Cardiovascular disease, n(%)	111(17.9)	27(17.9)	20(12.7)	33(21.0)	31(19.9)	0.2
Cerebral vascular disease,n(%)	55(8.9)	11(7.3)	19(12.1)	15(9.6)	10(6.4)	0.2
Hypertension, n(%)	532(85.7)	133(88.1)	138(87.9)	129(82.2)	132(84.6)	0.3
Diabetes mellitus, n(%)	239(38.5)	48(31.8)	57(36.3)	60(38.2)	74(47.4)	0.03
Hyperlipidemia, n(%)	272(43.8)	66(43.7)	77(49.0)	65(41.4)	64(41.0)	0.4
CKD cause, n(%)						
Chronic glomerulonephritis	232(37.4)	57(37.7	55(35.0)	61(38.9)	59(37.8)	0.6
Diabetic nephropathy	180(29)	41(27.2)	41(26.1)	47(29.9)	51(32.7)*	
Others	209(33.7)	53(35.1)	61(38.9)	49(31.2)	46(29.5)	
CKD stage 3 n(%)	146(23.5)	45(29.8)	37(23.6)	37(23.6)	27(17.3)	0.003
4 n(%)	243(39.1)	62(41.1)	72(45.9)	57(36.3)	52(33.3)	
5 n(%)	232(37.4)	44(29.1)	48(30.6)	63(40.1)	77(49.4)	
Medications						
Calcium channel blocker, n (%)	341(54.9)	80(53.0)	89(56.7)	78(49.7)	94(60.3)	0.3
β-blocker, n (%)	147(23.7)	26(17.2)	28(17.8)	45(28.7)	48(30.8)* ^#^	0.005
ACEI/ARB, n (%)	353(56.8)	89(58.9)	90(57.3)	85(54.1)	89(57.1)	0.8
Statin, n(%)	170(27.4)	40(26.5)	47(29.9)	40(25.5)	43(27.6)	0.8
Laboratory parameters						
Blood urea nitrogen (mg/dl)	41.1(30.0,60.0)	34.9(26.9,52.8)	38.7(29.5,55.1)	41.5(30.9,60.1)	50.6(35.5,66.1)* ^#^	<0.001
Creatinine (mg/dl)	2.9(2.1,5.0)	2.8(1.9,4.4)	2.8(2.0,4.6)	3.0(2.2,5.6)	3.6(2.3,5.6)	0.005
eGFR (ml/min/1.73 m^2^)	21.8±12.6	24.7±13.9^†^	22.7±11.8	20.7±12.3*	18.9±11.9* ^#^	<0.001
Fasting sugar (g/dl)	101(92,117)	99(91,111)	100(94,119)	100(91,117)	102(92,126)* ^†^	0.3
Glycated hemoglobin (%)	5.8(5.5,6.7)	5.7(5.4,6.4)	5.9(5.6,6.8)	5.7(5.4,6.4)	6.1(5.5,7.2)* ^†^	0.01
Hemoglobin (g/dl)	10.9±2.1	11.7±2.1^#†^	11.0±2.1*	10.7±2.1*	10.4±1.9* ^#^	<0.001
Albumin (g/dl)	4.1(3.9,4.3)	4.2(4.0,4.4)^†^	4.2(3.9,4.3)^†^	4.0(3.8,4.3)* ^#^	4.0(3.7,4.2)* ^#^	<0.001
Phosphate (mg/dl)	4.1(3.6,4.8)	4.0(3.5,4.6)	4.0(3.6,4.6)	4.1(3.7,4.8)	4.3(3.8,5.1)	0.02
Calcium (mg/dl)	8.9±0.6	9.1±0.6^†^	9.0±0.6	8.8±0.6*	8.8±0.8*	<0.001
Uric acid (mg/dl)	7.6±1.9	7.5±1.9	7.6±1.8	7.4±1.5	7.8±2.1	0.2
Cholesterol (mg/dl)	187±45	192±47	190±40	182±45	188±46	0.2
Triglyceride (mg/dl)	115(78,173)	115(78,181)	112(82,172)	108(76,162)	126(79,181)	0.6
hsCRP (mg/L)	1.6(0.6,4.2)	1.4(0.7,3.5)	1.5(0.5,3.7)	1.5(0.6,3.5)	2.2(0.8,6.5)* ^# †^	0.03
Parathyroid hormone (pg/ml)	72(37,157)	62(36,142)	63(35,120)	76(40,190)	106(33,191)	0.2
Urine protein-creatinine ratio >1 g/g n (%)	275(49.3)	57(41.0)	66(46.8)	70(49.6)	82(59.9)* ^#^	0.01

Data are expressed as number (percentage) for categorical variables and mean±SD or median (25^th^, 75^th^ percentile) for continuous variables, as appropriate.

Conversion factors for units: eGFR in mL/min/1.73 m^2^ to mL/s/1.73 m^2^, ×0.01667 ; hemoglobin in g/dL to g/L, ×10; albumin in g/dL to g/L, ×10; calcium-phosphate product in mg^2^/dL^2^ to mmol^2^/L^2^, ×0.0806; cholesterol in mg/dL to mmol/L, ×0.02586; triglyceride in mg/dL to mmol/L, ×0.01129; uric acid in mg/dL toµmol/L, ×59.48.

Abbreviations: CKD, chronic kidney disease; ECW, extracellular water; ICW, intracellular water; TBW, total body water; ACEI, angiotensin converting enzyme inhibitors; ARB, angiotensin II receptor blockers; eGFR, estimated glomerular filtration rate; hsCRP, high-sensitivity C-reactive protein.

*P<0.05 compared with quartile 1; ^#^ *P*<0.05 compared with quartile 2; ^†^ *P*<0.05 compared with quartile 3.

a Angiopoietin-2 quartile cut at 1494.1, 1948.8, and 2593.1 pg/ml.

## Results

### Characteristics of Entire Cohort

A total of 621 participants with CKD stages 3 to 5 were analyzed (mean eGFR 21.8 ml/min/1.73 m^2^, 146 in stage 3, 243 in stage 4, 232 in stage 5). The mean age was 65.3±12.7 years and 55.4% were male. Table 1 shows the baseline clinical characteristics stratified by quartiles of Ang-2, divided at 1494.1, 1948.8, and 2593.1 pg/ml. Of all patients, 532 (85.7%) were hypertensive and 239(38.5%)were diabetic mellitus. Pre-existing and documented heart disease and cerebral vascular disease were noted in 111(17.9%) and 55(8.9%) of patients respectively. The proportion of diabetes and β-blocker, serum blood urea nitrogen, phosphate and high-sensitivity C-reactive protein levels, and urine protein-creatinine ratio increased and eGFR, serum hemoglobin, calcium, and albumin levels decreased with Ang-2 quartiles.

**Table 2:** Renal outcome of all subjects stratified by Angiopoietin-2 quartile.

	Entire Cohort N = 621	Angiopoietin-2^a^				
		Quartile 1 N = 151	Quartile 2 N = 157	Quartile 3 N = 157	Quartile 4 N = 156	P-trend
Follow-up time (month)	38.2±26.3	43.8±28.1	38.0±25.9	35.7±26.0	35.5±24.6	0.02
No. of SCr measurement	17 (9,27)	18(8, 27)	15(9,23)	15(9,24)	20(12,28)	0.01
Doubling creatinine (n,%)	165(26.6)	35(23.2)	37(23.6)	45(28.7)	48(30.8)	0.3
eGFR decline (mL/min/1.73 m^2^/year)	−1.6(−3.3,−0.4)	−1.3(−2.8,0.1)	−1.8(−3.6,−0.9)	−1.8(−3.8,−0.6)	−1.5(−3.1,−0.2)	0.01
Commencing dialysis (n,%)	224(36.1)	45(29.8)	43(27.4)	59(37.6)	77(49.4)	<0.001

Data are expressed as number (percentage) for categorical variables and median (25^th^, 75^th^ percentile) for continuous variables, as appropriate.

Conversion factors for units: eGFR in mL/min/1.73 m^2^ to mL/s/1.73 m^2^, ×0.01667.

Abbreviations: eGFR, estimated glomerular filtration rate.

a Angiopoietin-2 quartile cut at 1494.1, 1948.8, and 2593.1 pg/ml.

### Ang-2 and composite outcomes, either commencing dialysis or doubling creatinine

**Figure 1:**
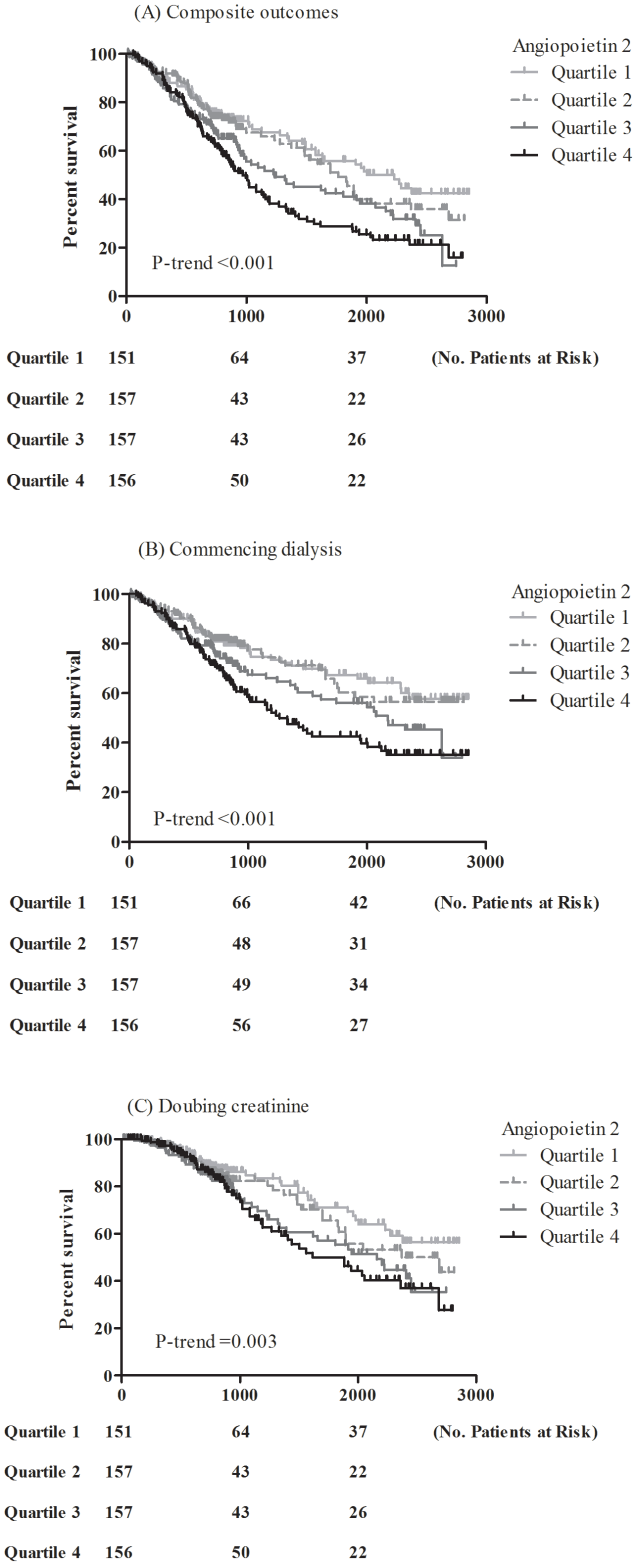
Kaplan-Meier survival curve for composite outcomes, either commencing dialysis or doubling creatinine of all subjects stratified by angiopoietin-2 quartile.

Over a mean follow-up period of 38.2±26.3 months, 224 patients (36.1%) progressed to commencing dialysis (198 hemodialysis and 26 peritoneal dialysis, Table 2). Seventy-one (11.4%) had mortality before reaching commencing dialysis. 18 (2.9%) were lost to follow-up (the mean follow-up period: 19.7±10.9 months), and no significant difference of proportion from quartile 1 to quartile 4 was found. A stepwise increase in the proportion of commencing dialysis from quartile 1 to quartile 4 was found (P-trend <0.001). Of all subjects, 165 (26.6%) reached doubling creatinine during follow-up period, but there was no significant difference among Ang-2 quartiles. Kaplan-Meier survival curve showed a significant correlation between quartiles of Ang-2 and composite outcomes, either commencing dialysis or doubling creatinine (Figure 1). Table 3 presents the longitudinal associations between stepwise increases in Ang-2 levels and composite outcomes, either commencing dialysis or doubling creatinine. The unadjusted hazard ratio (HR) of composite outcomes was 1.96 (95% Confidence interval (CI): 1.43–2.69) for subjects of quartile 4 compared with those of quartile 1. The adjusted HR of composite outcomes was 1.53 (95% CI: 1.06–2.23) for subjects of quartile 4 compared with those of quartile 1. The longitudinal association between composite outcomes and stepwise increases in Ang-2 levels (P-trend  = 0.03).

**Table 3:** The adjusted risks for composite outcomes, either commencing dialysis or doubling creatinine according to Angiopoietin-2 quartile.

Angiopoietin-2^a^	Composite outcomes		Commencing dialysis		Double creatinine	
	Hazard ratio (95% Cl)	P-value	Hazard ratio (95% Cl)	P-value	Hazard ratio (95% Cl)	P-value
Quartile 1	Reference		Reference		Reference	
Quartile 2	1.42(0.95–2.13)	0.08	1.50(0.92–2.44)	0.01	1.43(0.83–2.47)	0.1
Quartile 3	1.54(1.05–2.27)	0.02	1.73(1.10–2.71)	0.02	1.47(0.87–2.48)	0.1
Quartile 4	1.53(1.06–2.23)	0.02	1.85(1.20–2.85)	0.02	1.47(0.87–2.45)	0.1

Abbreviations: CI, Confidence Interval; eGFR, estimated glomerular filtration rate.

Adjusted model: age, sex, cardiovascular disease, diabetes mellitus, angiotensin converting enzyme inhibitors/angiotensin II receptor blockers use, estimated glomerular filtration rate, hemoglobin, serum calcium and cholesterol levels, log serum albumin and phosphate, and urine protein-creatinine ratio cut at 1 g/g.

a Angiopoietin-2 quartile cut at 1494.1, 1948.8, and 2593.1 pg/ml.

The unadjusted risk for commencing dialysis increased 2 fold (HR: 2.01, 95% CI: 1.39–2.90) for subjects of quartile 4 compared with those of quartile 1. The adjusted risk for commencing dialysis increased 85% (HR: 1.85, 95% CI: 1.20–2.85) for subjects of quartile 4 compared with those of quartile 1. The longitudinal association between commencing dialysis and stepwise increases in Ang-2 levels (P-trend  = 0.005). The unadjusted risk for doubling creatinine increased 89% (HR: 1.89, 95% CI: 1.21–2.93) for subjects of quartile 4 compared with those of quartile 1. However, there was no significant association of doubling creatinine with Ang-2 quartiles in adjusted model.

### Ang-2, rapid decline in renal function and change in eGFR

Eighty-five subjects (13.9%) had rapid decline in renal function. Patients of progressive decline in renal function (eGFR slope greater than 5 ml/min/1.73 m^2^/yr) were more likely to have higher level of Ang-2 than those of non-progressive decline in renal function (eGFR slope less than 5 ml/min/1.73 m^2^/yr) (median: 1944.9 *v.s.* 2066.0 pg/ml, P = 0.01). No significant different in baseline eGFR was found between the two groups. The adjusted risk for rapid decline in renal function increased 2.9 folds (OR: 2.96, 95% CI: 1.13–7.76) for subjects of quartile 4 compared with those of quartile 1 (Table 4). Table 4 also shows the effect of study group on the change in eGFR in the linear mixed-effects model. The highest quartile of Ang-2 was associated with a significant decrease in eGFR over time as compared with the lowest quartile of Ang-2 (unstandardized coefficient β = −1.73, 95% CI: −3.34,−0.11, P = 0.03).

**Table 4:** The adjusted risks for rapid decline in renal function and change of eGFR according to Angiopoietin-2 quartile.

Angiopoietin-2^a^	Rapid decline in renal function		Change of eGFR	
	Odds ratio (95% Cl)	P-value	Unstandardized coefficient β^b^ (95% Cl)	P-value
Quartile 1	Reference		Reference	
Quartile 2	3.40(1.32–8.75)	0.01	−1.03(−2.63,0.57)	0.1
Quartile 3	3.04(1.18–7.82)	0.02	−1.66(−3.25,−0.07)	0.03
Quartile 4	2.96(1.13–7.76)	0.02	−1.73(−3.33,−0.11)	0.03

Abbreviations: CI, Confidence Interval; eGFR, estimated glomerular filtration rate.

Adjusted model: age, sex, cardiovascular disease, diabetes mellitus, angiotensin converting enzyme inhibitors/angiotensin II receptor blockers use, estimated glomerular filtration rate, hemoglobin, serum calcium and cholesterol levels, log serum albumin and phosphate, , and urine protein-creatinine ratio cut at 1 g/g.

a Angiopoietin-2 quartile cut at 1494.1, 1948.8, and 2593.1 pg/ml.

b β expressed as ml/min/1.73 m^2^/year in eGFR.

## Discussion

To our knowledge, this study is the first to evaluate the association of Ang-2 with adverse renal outcome in patients with stages 3–5 CKD over an observation period of 3 years. Ang-2 is associated with composite renal outcomes, either commencing dialysis or doubling creatinine after adjustment of baseline renal function and associated risk factors. Patients with quartile 3 or more of Ang-2 have more than 1.7 and 3.0-fold increase in risk for commencing dialysis and rapid decline in renal function respectively. Additionally, the linear mixed-effects model shows a more rapid decrease in eGFR over time in patients with quartile 3 or more of Ang-2 than those with quartile 1 of Ang-2. CKD patients with quartile 3(1948.8 pg/ml) or more of Ang-2 are more likely to reach the plateau of adverse renal outcome.

With regard to glomerular diseases, studies by Belinda et al. [22] pointed out that increased glomerular expression of Ang-2 would tend to antagonize Ang-1-induced Tie-2 activation and destabilize capillaries and glomerular endothelia in podocin/Ang-2 transgene mice. There were significant increases in albuminuria and glomerular endothelial apoptosis, with significant decreases of both nephrin proteins and vascular endothelial growth factor A (VEGF-A), which were critical for maintenance of glomerular endothelia and glomerular filtration barrier integrity [23]–[25]. Besides, Seron et al. [26] indicated a chronic loss of renal interstitial capillaries in human nephropathy, and Futrakul et al. [27] suggested that an “anti-angiogenic environment” may exist in long-standing nephropathies. In clinical views, accumulating evidence shows that circulating Ang-2 is inversely related to eGFR and increases with advanced CKD and Ang-2 level is still increasing even after entering maintenance of dialysis [17]. However, little is known about a clinical relationship between Ang-2 and adverse renal outcome. The present study identifies that increased circulating Ang-2 is associated with risks for commencing dialysis and rapid decline in renal function in patients not on dialysis, and Ang-2 is an independent predictor of adverse renal outcome in CKD cohort.

Consistent with reports by Chang et al. [19], our results also showed a significant association of circulating Ang-2 with hypoalbuminemia and high-sensitivity C-reactive protein (hs-CRP), as indicators of malnutrition-inflammation. Fiedler et al. identified Ang-2 as an autocrine regulator of endothelial cell inflammatory responses and Ang-2 acts as a switch of vascular responsiveness exerting a permissive role for the activities of proinflammatory cytokines [11]. Ang-2 serves the link between angiogenic and inflammatory pathway, Ang-2 signaling between cellular elements in renal fibrosis, including endothelial cells, pericytes, myofibroblasts, and macrophages [23]. Meanwhile, CKD has been regarded as a disease with persistent and low-grade inflammation, and our previous study indicated that inflammation is an independent predictor of rapid decline in renal function in CKD cohort [28]. Hence, there might be an interaction among Ang-2, inflammation and rapid decline in renal function. The findings of our subgroup analysis show that elevated Ang-2 is independently associated with risk for maintenance dialysis in subjects of less than the median of hsCRP (1.56 mg/l; HR for log Ang-2: 4.80, 95%CI: 1.68–13.76). In addition, there is a significant association between Ang-2 and maintenance dialysis in subjects of serum albumin above 3.5 g/dl (HR for log Ang-2: 2.15, 95%CI: 1.05–4.38). Thus, circulating Ang-2 is possibly a significant risk factor for adverse renal outcome independent of malnutrition-inflammation.

Increased endothelial Ang-2 secretion is stimulated by exogenous stimuli such as angiotensin II, tumor necrosis factor-α, hypoxia, and reactive oxygen species, which are characteristics in CKD progression [5]. Endothelial injury in glomerular vasculature may induce endothelial Ang-2 secretion, and meanwhile, increased Ang-2 may lead to glomerular albuminuria through endothelial injury. It is difficult to evaluate whether increased Ang-2 is a cause or consequence of CKD progression [5]. Because of this complicated interaction among Ang-2, traditional risk factors, and CKD, we adjusted associated risk factors for rapid decline in renal function in multivariate analysis. We also performed subgroup analysis in different CKD stages, but the results did not consistent at all (Figure S1–S3). It is probably related to relative small number of subgroup and short observation period. We need further study to evaluate whether these risk factors are modifiers or confounders in association of Ang-2 and commencing dialysis. Besides, to consider the influence of competing risk of death on commencing dialysis, we performed further analysis, and the results are consistent (HR for quartile 4 compared with quartile 1: 1.85 (95% CI: 1.20–2.86). Our findings show a strong association of Ang-2 and adverse renal outcome and emphasize its importance as a predictor in CKD cohort.

Despite exogenous stimuli, Ang-2 was excreted by factors influencing the exocytosis of Weibel-Palade body, including thrombin, histamine, serotonin, vascular endothelial growth factor (VEGF) and epinephrine [29]. Therefore, it could be influenced by medication. In this study, no significant difference of medication was found among Ang-2 quartiles except for β-blocker. In post hoc multiple comparisons, subjects of Ang-2 quartile 4 had higher proportion of using β-blocker than those of Ang-2 quartile 1 and 2. The reason might be related to blood pressure control or arrhythmia. The present study has a limitation that we did not record blood pressure and arrhythmia at enrollment. In late CKD, patients usually need three or more kinds of anti-hypertension medicines to keep adequate blood pressure and use β-blocker for arrhythmia. It is probably the reason for the different proportion of β-blocker usage. Contrarily, β-blocker promotes cardiac angiogenesis in heart failure via activation of VEGF signaling pathway [30]. Hence, higher level of Ang-2 in late CKD patients may be partially related to long term usage of β-blocker. We also add β-blocker usage into multivariate analysis, and Ang-2 is still associated with adverse renal outcome. Further study is needed to investigate the relationship of ang-2 and β-blocker.

Ang-2 is usually elevated in diabetes and associated with endothelial dysfunction, which leads to microvascular and macrovascular complications [9], . The pathophysiological mechanisms by which Ang-2 participates in rapid decline in renal function are complicated and include various pathways, such as arterial stiffness or oxidative stress [17], [19]. Our results show an association of Ang-2 quartiles with the proportion of diabetes, but no correlation between Ang-2 quartiles and glycated hemoglobin in patients with integrated CKD care program. Although average sugar level is under strict control, some patients still reach adverse renal outcome. Accumulating evidence indicates that strict glycemic control might not be enough to prevent rapid decline in renal function in late CKD [33]. It is probably that Ang-2 is associated with adverse renal outcome beyond the effects of diabetes. Additionally, patients with cardiovascular disease, cerebrovascular disease or hypertension are more likely to have higher circulating Ang-2 level [10], [11], [13], [34]. Ang-2 has been associated with cardiovascular markers, such as cell adhesion molecules and inflammation [35], and increases endothelial apoptosis, enhances myocardial microvascular inflammation, and promotes cardiac fibrosis [31]. Although there was no different proportion of cardiovascular disease, cerebrovascular disease and hypertension in Ang-2 quartiles at baseline in our cohort, Ang-2 might be probably associated with cardiovascular events in the future. Thus, further study is needed to evaluate the relationship between Ang-2 and cardiovascular outcome.

On the other hand, previous study reported a significant correlation between Ang-2 and asymmetric dimethylarginine (ADMA), as the nitric oxide (NO) synthase inhibitor [17]. CKD has been regarded as a NO-deficient state, and the oxidative stress leads to not only renal function decline, but also adverse cardiovascular sequelae [36], [37]. Thus, ADMA is not only a uremic toxin, but also a strong marker of endothelial dysfunction and atherosclerosis [38]. Sascha et al. speculated that the increased Ang-2 levels might reveal excess Weibel-Palade body exocytosis as a consequence of decreased NO bioavailability in the presence of high ADMA levels [17]. Although further in vivo and in vitro studies are needed to evaluate the interaction between Ang-2 and NO bioavailability, it could possibly explain one of the potential mechanisms responsible for the association between Ang-2 and adverse renal outcome.

This study has some limitations that must be considered. The major uncertainty is whether circulating Ang-2 is biologically active in CKD patients. The biological implication of Ang-2 changes in the range observed in our patients is still unknown. Besides, Ang-2 was measured once at enrollment. The effect of the time-varying Ang-2 levels might be underestimated. Additionally, the mechanism contributing to the association between increased circulating Ang-2 and rapid decline in renal function has not been well-explored. Further study is needed to investigate the pathogenic link between Ang-2 and rapid decline in renal function.

In conclusion, our study demonstrates that elevated circulating Ang-2 is associated with increased risks for adverse renal outcome in stages 3–5 CKD patients. Future studies will be necessary to evaluate the pathogenic role of Ang-2 in renal progression, and to establish the beneficial renal function by targeting Ang-2.

## Supporting Information

Figure S1 **Adjusted hazard ratios (HRs) of commencing dialysis for Angiopoietin-2 (Ang-2) quartile 4 compared with Ang-2 quartile 1 in CKD stages 3–5 subjects stratified by proteinuria, high sensitivity c-reactive protein (hsCRP), serum albumin and angiotensin converting enzyme inhibitors (ACEI)/angiotensin II receptor blockers (ARB) usage.** Ratios were adjusted for age, sex, cardiovascular disease, diabetes mellitus, ACEI/ARB usage, estimated glomerular filtration rate, hemoglobin, serum calcium and cholesterol levels, log serum albumin and phosphate, and urine protein-creatinine ratio cut at 1 g/g. The median values of serum albumin and hsCRP are 3.8 g/dl and 1.5 mg/l respectively.
(TIF)

Figure S2 **Adjusted hazard ratios (HRs) of commencing dialysis for Angiopoietin-2 (Ang-2) quartile 4 compared with Ang-2 quartile 1 in CKD stages 3–4 subjects stratified by proteinuria, high sensitivity c-reactive protein (hsCRP), serum albumin and angiotensin converting enzyme inhibitors (ACEI)/angiotensin II receptor blockers (ARB) usage.** Ratios were adjusted for age, sex, cardiovascular disease, diabetes mellitus, ACEI/ARB usage, estimated glomerular filtration rate, hemoglobin, serum calcium and cholesterol levels, log serum albumin and phosphate, and urine protein-creatinine ratio cut at 1 g/g. The median values of serum albumin and hsCRP are 3.8 g/dl and 1.5 mg/l respectively.
(TIF)

Figure S3 **Adjusted hazard ratios (HRs) of commencing dialysis for Angiopoietin-2 (Ang-2) quartile 4 compared with Ang-2 quartile 1 in CKD stage 5 subjects stratified by proteinuria, high sensitivity c-reactive protein (hsCRP), serum albumin and angiotensin converting enzyme inhibitors (ACEI)/angiotensin II receptor blockers (ARB) usage.** Ratios were adjusted for age, sex, cardiovascular disease, diabetes mellitus, ACEI/ARB usage, estimated glomerular filtration rate, hemoglobin, serum calcium and cholesterol levels, log serum albumin and phosphate, and urine protein-creatinine ratio cut at 1 g/g. The median values of serum albumin and hsCRP are 3.8 g/dl and 1.5 mg/l respectively.
(TIF)

## References

[1] Nugent RA, Fathima SF, Feigl AB, Chyung D (2011) The burden of chronic kidney disease on developing nations: a 21st century challenge in global health. Nephron Clin Pract 118: 269–277.. 10.1159/000321382 21212690

[2] Chang FC, Lin SL (2013) The role of angiopoietin-2 in progressive renal fibrosis. J Formos Med Assoc 112: 175–176.. 23537862 10.1016/j.jfma.2012.07.028

[3] Brindle NP, Saharinen P, Alitalo K (2006) Signaling and functions of angiopoietin-1 in vascular protection. Circ Res 98: 1014–1023.. 16645151 10.1161/01.RES.0000218275.54089.12 PMC2270395

[4] Fam NP, Verma S, Kutryk M, Stewart DJ (2003) Clinician guide to angiogenesis. Circulation 108: 2613–2618.. 14638526 10.1161/01.CIR.0000102939.04279.75

[5] Fiedler U, Augustin HG (2006) Angiopoietins: a link between angiogenesis and inflammation. Trends Immuno 27: 552–558.. 10.1016/j.it.2006.10.004 17045842

[6] Fiedler U, Scharpfenecker M, Koidl S, Hegen A, Grunow V, et al (2004) The Tie-2 ligand angiopoietin-2 is stored in and rapidly released upon stimulation from endothelial cell Weibel-Palade bodies. Blood 103: 4150–4156.. 14976056 10.1182/blood-2003-10-3685

[7] Huang YQ, Li JJ, Hu L, Lee M, Karpatkin S (2002) Thrombin induces increased expression and secretion of angiopoietin-2 from human umbilical vein endothelial cells. Blood 99: 1646–1650.. 11861279 10.1182/blood.v99.5.1646

[8] Fiedler U, Reiss Y, Scharpfenecker M, Grunow V, Koidl S, et al (2006) Angiopoietin-2 sensitizes endothelial cells to TNF-alpha and has a crucial role in the induction of inflammation. Nat Med 12: 235–239.. 16462802 10.1038/nm1351

[9] Lim HS, Lip GY, Blann AD (2005) Angiopoietin-1 and angiopoietin-2 in diabetes mellitus: relationship to VEGF, glycaemic control, endothelial damage/dysfunction and atherosclerosis. Atherosclerosis 180: 113–118.. 15823283 10.1016/j.atherosclerosis.2004.11.004

[10] Nadar SK, Blann A, Beevers DG, Lip GY (2005) Abnormal angiopoietins 1&2, angiopoietin receptor Tie-2 and vascular endothelial growth factor levels in hypertension: relationship to target organ damage [a sub-study of the Anglo-Scandinavian Cardiac Outcomes Trial (ASCOT)]. J Intern Med 258: 336–343.. 16164572 10.1111/j.1365-2796.2005.01550.x

[11] Chong AY, Caine GJ, Freestone B, Blann AD, Lip GY (2004) Plasma angiopoietin-1, angiopoietin-2, and angiopoietin receptor tie-2 levels in congestive heart failure. J Am Coll Cardiol 43: 423–428.. 15013125 10.1016/j.jacc.2003.08.042

[12] David S, Kumpers P, Hellpap J, Horn R, Leitolf H, et al (2009) Angiopoietin-2 and cardiovascular disease in dialysis and kidney transplantation. Am J Kidney Dis 53: 770–778.. 19268412 10.1053/j.ajkd.2008.11.030

[13] Lee KW, Lip GY, Blann AD (2004) Plasma angiopoietin-1, angiopoietin-2, angiopoietin receptor tie-2, and vascular endothelial growth factor levels in acute coronary syndromes. Circulation 110: 2355–2360.. 15302795 10.1161/01.CIR.0000138112.90641.7F

[14] Parikh SM, Mammoto T, Schultz A, Yuan HT, Christiani D, et al (2006) Excess circulating angiopoietin-2 may contribute to pulmonary vascular leak in sepsis in humans. PLoS Med 3: e46.. 16417407 10.1371/journal.pmed.0030046 PMC1334221

[15] Kumpers P, Lukasz A, David S, Horn R, Hafer C, et al (2008) Excess circulating angiopoietin-2 is a strong predictor of mortality in critically ill medical patients. Crit Care 12: R147.. 19025590 10.1186/cc7130 PMC2646310

[16] Kumpers P, Hafer C, David S, Hecker H, Lukasz A, et al (2010) Angiopoietin-2 in patients requiring renal replacement therapy in the ICU: relation to acute kidney injury, multiple organ dysfunction syndrome and outcome. Intensive Care Med 36: 462–470.. 19956923 10.1007/s00134-009-1726-7

[17] David S, Kumpers P, Lukasz A, Fliser D, Martens-Lobenhoffer J, et al (2010) Circulating angiopoietin-2 levels increase with progress of chronic kidney disease. Nephrol Dial Transplant 25: 2571–2576.. 20179005 10.1093/ndt/gfq060

[18] David S, John SG, Jefferies HJ, Sigrist MK, Kumpers P, et al (2012) Angiopoietin-2 levels predict mortality in CKD patients. Nephrol Dial Transplant 27: 1867–1872.. 21976741 10.1093/ndt/gfr551

[19] Chang FC, Lai TS, Chiang CK, Chen YM, Wu MS, et al (2013) Angiopoietin-2 is associated with albuminuria and microinflammation in chronic kidney disease. PloS One 8: e54668.. 23469160 10.1371/journal.pone.0054668 PMC3585725

[20] Levey AS, Bosch JP, Lewis JB, Greene T, Rogers N, et al (1999) A more accurate method to estimate glomerular filtration rate from serum creatinine: a new prediction equation. Modification of Diet in Renal Disease Study Group. Ann Intern Med 130: 461–470.. 10075613 10.7326/0003-4819-130-6-199903160-00002

[21] Levin A, Stevens PE (2014) Summary of KDIGO 2012 CKD Guideline: behind the scenes, need for guidance, and a framework for moving forward. Kidney Int 85: 49–61.. 24284513 10.1038/ki.2013.444

[22] Davis B, Dei Cas A, Long DA, White KE, Hayward A, et al (2007) Podocyte-specific expression of angiopoietin-2 causes proteinuria and apoptosis of glomerular endothelia. J Am Soc Nephrol 18: 2320–2329.. 17625119 10.1681/ASN.2006101093

[23] Woolf AS, Gnudi L, Long DA (2009) Roles of angiopoietins in kidney development and disease. J Am Soc Nephrol 20: 239–244.. 18799719 10.1681/ASN.2008020243

[24] Tryggvason K, Patrakka J, Wartiovaara J (2006) Hereditary proteinuria syndromes and mechanisms of proteinuria. N Engl J Med 354: 1387–1401.. 16571882 10.1056/NEJMra052131

[25] Eremina V, Sood M, Haigh J, Nagy A, Lajoie G, et al (2003) Glomerular-specific alterations of VEGF-A expression lead to distinct congenital and acquired renal diseases. J Clin Invest 111: 707–716.. 12618525 10.1172/JCI17423 PMC151905

[26] Seron D, Alexopoulos E, Raftery MJ, Hartley B, Cameron JS (1990) Number of interstitial capillary cross-sections assessed by monoclonal antibodies: relation to interstitial damage. Nephrol dial transplant 5: 889–893.. 2128387 10.1093/ndt/5.10.889

[27] Futrakul N, Butthep P, Futrakul P (2008) Altered vascular homeostasis in chronic kidney disease. Clin Hemorheol Microcirc 38: 201–207.. 18239262

[28] Tsai YC, Hung CC, Kuo MC, Tsai JC, Yeh SM, et al (2012) Association of hsCRP, white blood cell count and ferritin with renal outcome in chronic kidney disease patients. PloS One 7: e52775.. 23300770 10.1371/journal.pone.0052775 PMC3534111

[29] Rondaij MG, Bierings R, Kragt A, van Mourik JA, Voorberg J (2006) Dynamics and plasticity of Weibel-Palade bodies in endothelial cells. Arterioscler Thromb Vasc Biol 26: 1002–1007.. 16469951 10.1161/01.ATV.0000209501.56852.6c

[30] Rengo G, Cannavo A, Liccardo D, Zincarelli C, de Lucia C, et al (2013) CVascular endothelial growth factor blockade prevents the beneficial effects of β-blocker therapy on cardiac function, angiogenesis, and remodeling in heart failure. Circ Heart Fail 6: 1259–67.. 24029661 10.1161/CIRCHEARTFAILURE.113.000329

[31] Chen JX, Zeng H, Reese J, Aschner JL, Meyrick B (2012) Overexpression of angiopoietin-2 impairs myocardial angiogenesis and exacerbates cardiac fibrosis in the diabetic db/db mouse model. Am J Physiol Heart Circ Physiol 302: 1003–1012.. 10.1152/ajpheart.00866.2011 PMC3322731 22180648

[32] Anuradha S, Mohan V, Gokulakrishnan K, Dixit M (2010) Angiopoietin-2 levels in glucose intolerance, hypertension, and metabolic syndrome in Asian Indians (Chennai Urban Rural Epidemiology Study-74). Metabolism Clinical and Experimental 59: 774–779.. 19913849 10.1016/j.metabol.2009.09.022

[33] Shurraw S, Hemmelgarn B, Lin M, Majumdar SR, Klarenbach, et al (2011) SAssociation between glycemic control and adverse outcomes in people with diabetes mellitus and chronic kidney disease: a population-based cohort study. Arch Intern Med 171: 1920–1927.. 22123800 10.1001/archinternmed.2011.537

[34] Cui X, Chopp M, Zacharek A, Ye X, Roberts C, et al (2011) Angiopoietin/Tie2 pathway mediates type 2 diabetes induced vascular damage after cerebral stroke. Neurobiol Dis 43: 285–292.. 21515377 10.1016/j.nbd.2011.04.005 PMC3096677

[35] Shroff RC, Price KL, Kolatsi-Joannou M, Todd AF, Wells D, et al (2013) Circulating angiopoietin-2 is a marker for early cardiovascular disease in children on chronic dialysis. PLOS one 8: e56273.. 23409162 10.1371/journal.pone.0056273 PMC3568077

[36] Wever R, Boer P, Hijmering M, Stroes E, Verhaar M, et al (1999) Nitric oxide production is reduced in patients with chronic renal failure. Arterioscler Thromb Vasc Biol 19: 1168–1172.. 10323766 10.1161/01.atv.19.5.1168

[37] Baylis C (2012) Nitric oxide synthase derangements and hypertension in kidney disease. Curr Opin Nephrol Hypertens 21: 1–6.. 22048724 10.1097/MNH.0b013e32834d54ca PMC3277934

[38] Kielstein JT, Zoccali C (2005) Asymmetric dimethylarginine: a cardiovascular risk factor and a uremic toxin coming of age? Am J Kidney Dis 46: 186–202. 16112037 10.1053/j.ajkd.2005.05.009

