# Supplementary figures and images for: Association of Angiopoietin-2 with Renal Outcome in Chronic Kidney Disease
Source: PLoS One. 2014 Oct 3;9(10):e108862. doi: 10.1371/journal.pone.0108862 (PMC4184837; doi:10.1371/journal.pone.0108862)

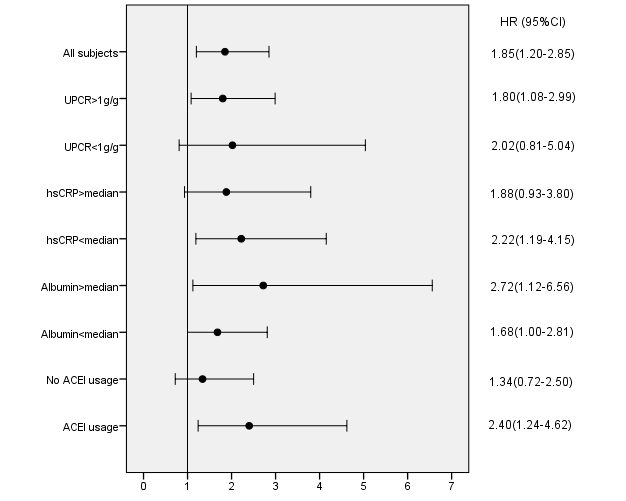

Supplement: Figure S1 — Adjusted hazard ratios (HRs) of commencing dialysis for Angiopoietin-2 (Ang-2) quartile 4 compared with Ang-2 quartile 1 in CKD stages 3–5 subjects stratified by proteinuria, high sensitivity c-reactive protein (hsCRP), serum albumin and angiotensin converting enzyme inhibitors (ACEI)/angiotensin II receptor blockers (ARB) usage. Ratios were adjusted for age, sex, cardiovascular disease, diabetes mellitus, ACEI/ARB usage, estimated glomerular filtration rate, hemoglobin, serum calcium and cholesterol levels, log serum albumin and phosphate, and urine protein-creatinine ratio cut at 1 g/g. The median values of serum albumin and hsCRP are 3.8 g/dl and 1.5 mg/l respectively. (TIF) [file pone.0108862.s001.tif]

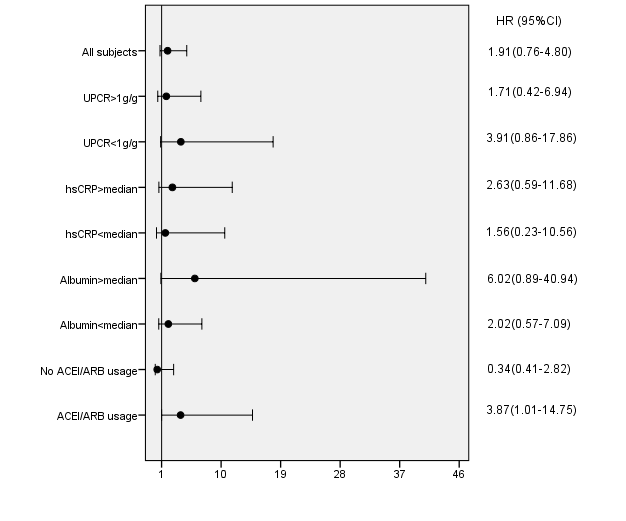

Supplement: Figure S2 — Adjusted hazard ratios (HRs) of commencing dialysis for Angiopoietin-2 (Ang-2) quartile 4 compared with Ang-2 quartile 1 in CKD stages 3–4 subjects stratified by proteinuria, high sensitivity c-reactive protein (hsCRP), serum albumin and angiotensin converting enzyme inhibitors (ACEI)/angiotensin II receptor blockers (ARB) usage. Ratios were adjusted for age, sex, cardiovascular disease, diabetes mellitus, ACEI/ARB usage, estimated glomerular filtration rate, hemoglobin, serum calcium and cholesterol levels, log serum albumin and phosphate, and urine protein-creatinine ratio cut at 1 g/g. The median values of serum albumin and hsCRP are 3.8 g/dl and 1.5 mg/l respectively. (TIF) [file pone.0108862.s002.tif]

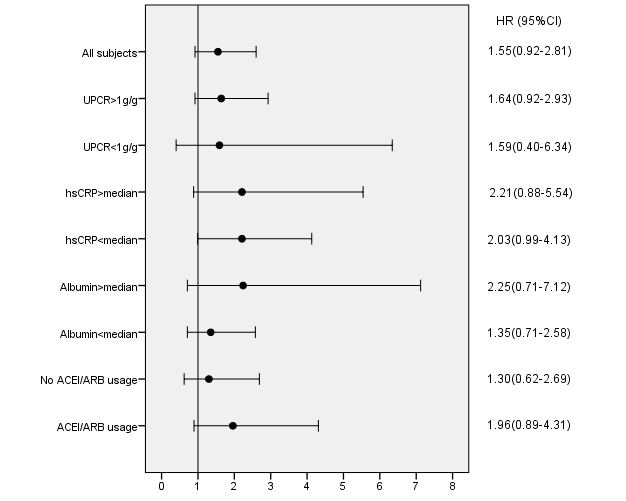

Supplement: Figure S3 — Adjusted hazard ratios (HRs) of commencing dialysis for Angiopoietin-2 (Ang-2) quartile 4 compared with Ang-2 quartile 1 in CKD stage 5 subjects stratified by proteinuria, high sensitivity c-reactive protein (hsCRP), serum albumin and angiotensin converting enzyme inhibitors (ACEI)/angiotensin II receptor blockers (ARB) usage. Ratios were adjusted for age, sex, cardiovascular disease, diabetes mellitus, ACEI/ARB usage, estimated glomerular filtration rate, hemoglobin, serum calcium and cholesterol levels, log serum albumin and phosphate, and urine protein-creatinine ratio cut at 1 g/g. The median values of serum albumin and hsCRP are 3.8 g/dl and 1.5 mg/l respectively. (TIF) [file pone.0108862.s003.tif]
